# Lipids and Fatty Acids of Nudibranch Mollusks: Potential Sources of Bioactive Compounds

**DOI:** 10.3390/md12084578

**Published:** 2014-08-19

**Authors:** Natalia V. Zhukova

**Affiliations:** 1 Institute of Marine Biology, Far East Branch, Russian Academy of Sciences, Vladivostok 690041, Russia; E-Mail: nzhukova35@list.ru; Tel.: +7-423-231-0937; Fax: +7-423-231-0900; 2 School of Biomedicine, Far Eastern Federal University, Vladivostok 690950, Russia

**Keywords:** mollusks, symbiotic bacteria, fatty acids, phospholipids

## Abstract

The molecular diversity of chemical compounds found in marine animals offers a good chance for the discovery of novel bioactive compounds of unique structures and diverse biological activities. Nudibranch mollusks, which are not protected by a shell and produce chemicals for various ecological uses, including defense against predators, have attracted great interest for their lipid composition. Lipid analysis of eight nudibranch species revealed dominant phospholipids, sterols and monoalkyldiacylglycerols. Among polar lipids, 1-alkenyl-2-acyl glycerophospholipids (plasmalogens) and ceramide-aminoethyl phosphonates were found in the mollusks. The fatty acid compositions of the nudibranchs differed greatly from those of other marine gastropods and exhibited a wide diversity: very long chain fatty acids known as demospongic acids, a series of non-methylene-interrupted fatty acids, including unusual 21:2∆7,13, and an abundance of various odd and branched fatty acids typical of bacteria. Symbiotic bacteria revealed in some species of nudibranchs participate presumably in the production of some compounds serving as a chemical defense for the mollusks. The unique fatty acid composition of the nudibranchs is determined by food supply, inherent biosynthetic activities and intracellular symbiotic microorganisms. The potential of nudibranchs as a source of biologically active lipids and fatty acids is also discussed.

## 1. Introduction

The molecular diversity of chemical compounds found in marine animals is the result of the evolution of the organisms and their unique physiological and biochemical adaptations and offers a good chance for the discovery of novel bioactive compounds with a variety of unique structures and diverse biological activities [1]. Marine mollusks have become the focus of many chemical studies aimed at isolating and identifying novel natural products [2]. Phylum Mollusca is the second largest phylum of animals. Nudibranch mollusks, which often are very colorful, are not protected by a shell and are named sea slugs, have attracted strong interest for their secondary metabolites, which are active in chemical defenses against predators [3]. These compounds exhibit a large variety of chemical structures [4,5] and have been shown to possess ichthyotoxic, feeding-deterrent and cytotoxic properties, to have antibacterial activity, to act as sexual pheromones [6] and are responsible for various bioactivities, such as antitumor, anti-inflammatory and antioxidant activities. Clearly, dietary sources contribute significantly to the chemical diversity of metabolites found in some mollusks [6]. However, their *de novo* biosynthesis has been reported for several mollusk species [7]. The secondary metabolites isolated from mollusks fall into a wide range of structural classes, with some compounds predominating in certain taxa. In the Gastropoda, terpenes dominate, whereas fatty acid derivatives are relatively uncommon [2].

Mollusks, as well as the invertebrates, in general, constitute a source of lipid bioactive compounds offering a variety of nutraceutical and pharmaceutical applications [2]. Among them, the omega-3 polyunsaturated fatty acids (PUFA), such as eicosapentaenoic acid, 20:5*n*-3, and docosahexaenoic acid, 22:6*n*-3, are known for their beneficial effects on human health [8]. These PUFA *n*-3 fatty acids are widely known for their capacities for cardioprotection; they reduce triacylglycerol and cholesterol levels and have anti-inflammatory and anticancer effects [9]. Numerous experiments on animals confirmed the cancer preventive properties of PUFA *n*-3 fatty acids from marine sources [9,10].

Some other marine lipids also show many potential bioactive properties. Monogalactosyldiacylglycerols and digalactosyldiacylglycerols from the marine microalga, *Nannochloropsis granulata*, have been reported to have a nitric oxide inhibitory activity [11]. The betaine lipid from microalgae *N. granulata*, diacylglyceryltrimethylhomoserine, shows a nitric oxide inhibitory activity, indicating a possible value as an anti-inflammatory agent [12]. The glycolipid, sulfoquinovosyl diacylglycerol, from red alga *Osmundaria obtusiloba* [13] and from brown alga *Sargassum vulgare* [14] exhibits a potent antiviral activity against herpes simplex virus type 1 and 2. This glycolipid from a brown alga, *Lobophora variegata*, possess a pronounced antiprotozoal activity [15]. Studies on glycosphingolipids from marine sponge *Axinyssa djiferi* proved their good antiplasmodial activity [16].

Although interest in the fatty acid composition of mollusks has not been abated, it has become increasingly obvious that phyla of marine invertebrates may be a source of unusual marine lipids, such as plasmalogens, phospholipids, glycolipids and diverse fatty acids.

The aim of the work was to fill a gap in the knowledge of the lipid biochemistry of mollusks. In particular, we consider data on the lipid of the nudibranchs (Mollusca, Gastropoda, Opisthobranchia, Nudibranchia). Herein, we report the investigation of the eight common species of nudibranchs with the use of the high-performance thin-layer chromatography (HPTLC), gas chromatography coupled with flame ionization detection (GC-FID) and gas chromatography coupled with mass spectrometry (GC-MS) methods to elucidate their lipid, phospholipid and fatty acid composition. A suggestion on the origin of the fatty acid variety in nudibranchs and their potential as bioactive compounds is also given.

## 2. Results and Discussion

### 2.1. Lipids and Phospholipids

**Figure 1:**
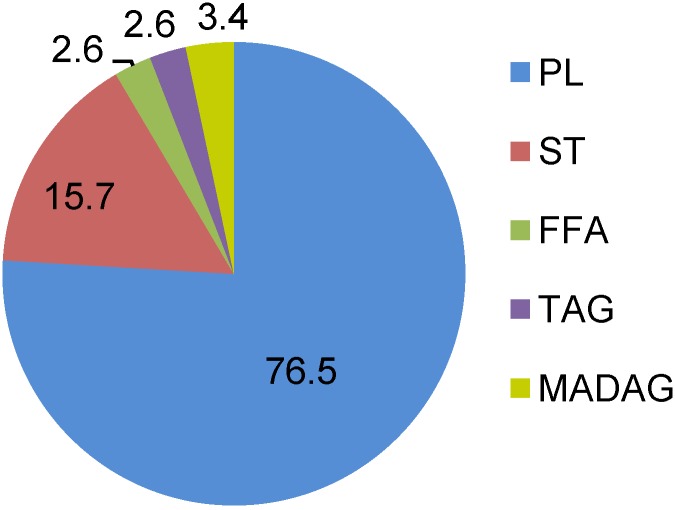
Lipid classes (% of total lipids) of nudibranchs. Results are expressed as the mean of eighth species (*n* = 8). PL, phospholipid; ST, sterol; FFA, free fatty acid; TAG, triacylglycerol; MADAG, monoalkyldiacylglycerol.

Lipids exert important biological functions as energy storage compounds, structural components of the cell membranes and as signaling molecules. The lipid content of the nudibranchs accounts for 14.2–21.4 mg·g^−1^ wet weight. The eight studied species of the nudibranchs appeared to have similar lipid compositions. According to this similarity in the lipid classes of these nudibranchs, the amounts of the lipid classes insignificantly vary depending on species and environmental conditions. Statistical analysis confirmed that lipid class values differed insignificantly among species. Hence, Figure 1 gives the average results for all studied species. The lipid composition of nudibranchs revealed that the major lipid class was phospholipids (PLs) and, to a lesser extent, sterols (STs) (13.5%–16.1% of total lipids). The PL concentration varied within a range from 73.8% in *Chromodoris geometrica* to 81.7% in *Glossodoris cincta*; this was much more than was found in other mollusks and invertebrates in total [17]. Triacylglycerols (TAGs), monoalkyldiacylglycerols (MADAGs) and free fatty acids (FFAs), which are the storage compounds of the cells, were minor components (2.6%, 3.4% and 2.6%, respectively). The detected distribution was similar to that found in two other tropical species of nudibranchs [18] and confirmed the high membrane phospholipids and low storage lipids in the tissues of these mollusks. The level of the neutral storage lipids is known to be species specific and depends mainly on the life history strategy and food availability [19].

The PL composition of the studied species was similar, with the dominance of phosphatidylcholine (PC) (up to 62.8% of total PL in *Chromodoris tinctoria*) and further, in descending order: phosphatidylethanolamine (PE), phosphatidylserine (PS), ceramide-aminoethylphosphonate (CAEP), phosphatidylinositol (PI) and diphosphatidylglycerol (DPG) (Table 1). The PL data obtained for the nudibranchs were different from those of other mollusks species by the elevated concentration of PC. The phosphonolipid, CAEP, is relatively abundant in some invertebrates, and it has been detected previously in freshwater and marine mollusks [20].

**Table 1:** Composition of phospholipids in the nudibranchs (mol%). Results are expressed as the mean ± SD of four replicates (*n* = 4). PC, phosphatidylcholine; PE, phosphatidylethanolamine; PS, phosphatidylserine; CAEP, ceramide-aminoethylphosphonate; PI, phosphatidylinositol; DPG, diphosphatidylglycerol.

	PC	PE	PS	CAEP	PI	DPG
*Chromodoris tinctoria*	60.9 ± 2.1	11.7 ± 0.9	12.5 ± 1.1	5.1 ± 0.4	6.5 ± 0.8	2.0 ± 0.6
*C. michaeli*	53.1 ± 2.1	21.4 ± 1.1	13.4 ± 0.8	5.6 ± 0.5	4.9 ± 0.6	1.7 ± 0.4
*C. geometrica*	53.8 ± 1.5	15.4 ± 0.9	12.6 ± 0.7	12.2 ± 1.1	3.7 ± 1.1	1.2 ± 0.3
*Chromodoris* sp.	51.2 ± 1.1	17.4 ± 1.3	14.5 ± 1.2	9.1 ± 1.9	5.0 ± 0.6	1.8 ± 0.4
*Glossodoris cincta*	56.1 ± 0.6	16.4 ± 1.4	15.4 ± 1.3	5.1 ± 0.7	4.2 ± 0.6	1.8 ± 0.3
*G. atromarginata*	53.5 ± 2.3	18.2 ± 1.9	12.2 ± 1.4	9.9 ± 1.7	5.1 ± 0.6	1.1 ± 0.2
*Risbecia tryoni*	49.6 ± 0.4	18.2 ± 1.1	13.8 ± 0.8	10.6 ± 1.1	4.6 ± 1.0	3.2 ± 0.8
*Platydoris* sp.	50.9 ± 2.0	21.2 ± 1.5	10.2 ± 0.8	8.0 ± 1.7	5.4 ± 0.5	2.7 ± 0.5

Marine invertebrates are known as a rich source of 1-alkenyl-2-acyl glycerophospholipids, commonly called plasmalogens [17,21]. Plasmalogens are particular phospholipids characterized by the presence of a vinyl ether bond at the C1 position of the glycerol skeleton. Plasmalogens are also ubiquitously found in animal cells. In mammals, the brain, heart, lymphocytes, spleen, macrophages and polymorphonuclear leukocytes contain the highest amount of plasmalogen-ethanolamine [22]. Two PLs, PE and PS, were represented as diacyl- and alkenyl-forms, and more than half of these aminophospholipids were plasmalogens (Table 2). 1-Alkenyl-2-acyl-PE made up 50.3%–65.1% of total PE; and 1-alkenyl-2-acyl-PS reached 47.1%–61.3% of total PS. The highest percentage of PE plasmalogens was found in *Risbecia tryoni*, accounting for 65.1% of total PE, and the PS plasmalogen contribution reached 61.3% of total PS in *Platydoris* sp. In contrast to many marine and freshwater mollusks, the nudibranchs contained PC only as a diacyl-form. Earlier, plasmalogens have been detected in PE, PS and PC in common edible mollusk species; the PE fraction is very often composed predominantly of the plasmalogens [23,24].

**Table 2:** Content of plasmalogens (1-alkenyl-2-acyl glycerophospholipids) in the nudibranchs. Expressed as the proportion of the plasmalogen forms relative to the whole of the same class forms; mean ± SD of four replicates (*n* = 4).

	1-Alkenyl-2-acyl-PE	1-Alkenyl-2-acyl-PS
*Chromodoris tinctoria*	60.4 ± 1.6	47.1 ± 2.1
*Chromodoris michaeli*	52.9 ± 2.4	51.1 ± 2.4
*Chromodoris geometrica*	58.8 ± 2.1	50.6 ± 1.7
*Chromodoris* sp.	59.2 ± 1.8	48.7 ± 1.1
*Glossodoris cincta*	61.1 ± 1.1	47.8 ± 1.5
*Glossodoris atromarginata*	60.7 ± 2.1	48.6 ± 1.2
*Risbecia tryoni*	65.1 ± 1.6	56.5 ± 1.0
*Platydoris* sp.	50.3 ± 1.8	61.3 ± 1.5

Serving as a structural component of the mammalian and invertebrate cell membrane, plasmalogens are widely distributed in excitable tissues, like heart and brain. Plasmalogens mediate the dynamics of the cell membrane. They provide storage for polyunsaturated fatty acids and can contribute to endogenous antioxidant activity, thus protecting cells from oxidative stress [25]. Plasmalogen phospholipids are suggested to be involved in signal transduction [26]. Plasmalogens are not only components of the plasma membrane and of lung surfactant, they serve as a reservoir for secondary messengers and may be also involved in membrane fusion, ion transport and cholesterol efflux. Low levels of these metabolites have trophic effects, but at a high concentration, they are cytotoxic and may be involved in allergic response, inflammation and trauma. Decreased levels of plasmalogens are associated with several neurological disorders, including Alzheimer’s disease, ischemia and spinal cord trauma [27].

### 2.2. Fatty Acids

**Figure 2:**
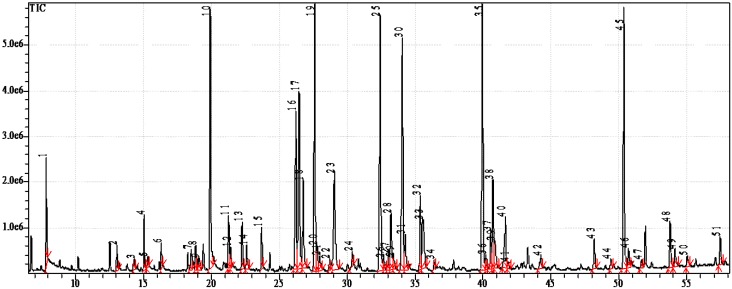
GC-MS chromatogram of 4,4-dimethyloxazoline derivatives from *Chromodoris michaeli*.

**Figure 3:**
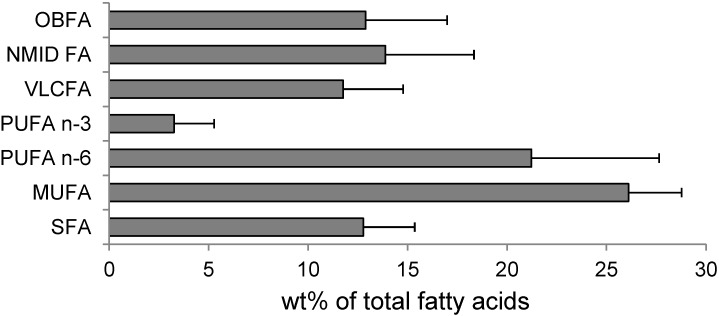
Distribution of fatty acids in nudibranch species. Results are expressed as the mean ± SD of eight studied species. OBFA, odd-chain and branched; NMID, non-methylene-interrupted dienoic; VLCFA, very long chain fatty acids.

The fatty acid profiles of the studied species were rather similar and differed only in their qualitative proportions of the fatty acids. Figure 2 shows the GC-MS chromatogram of the 4,4-dimethyloxazoline (DMOX) derivatives from the sea slug, *Chromodoris michaeli*. Table 3 reports the qualitative and quantitative data obtained, respectively, from GC-MS and GC-FID analyses. The components were eluted according their chain length and the degree of unsaturation in the chain on the MDN-5S capillary column. The chromatographic analyses allowed us to detect and identify about 50 individual fatty acids. The nudibranchs exhibited a wide diversity of fatty acids, including common saturated fatty acids (SFA) (8.6%–16.5% of total fatty acids), monounsaturated fatty acids (MUFA) (22.7%–31.2%) and polyunsaturated fatty acids (PUFA) (15.1%–31.4%), as well as non-methylene-interrupted dienoic fatty acids (NMID FA) (8.0%–21.5%), very long chain fatty acids (VLCFAs) (7.7%–16.6%) and odd-chain and branched fatty acids (5.0%–17.4%) (Figure 3).

**Figure 4:**
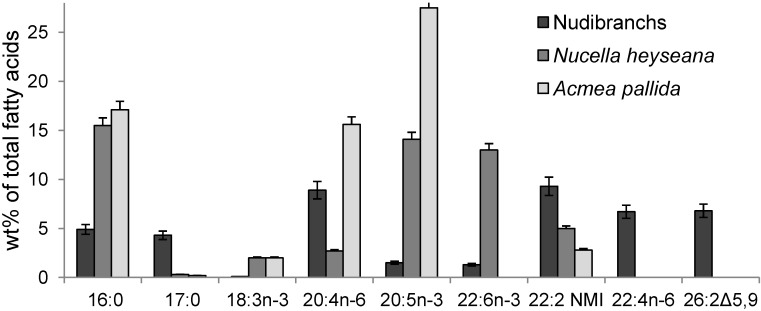
Fatty acid composition (wt%) of nudibranchs. Results are expressed as the mean ± SD of eight studied species.

Marine mollusks are generally characterized by the predominance of essential *n*-3 PUFA, mainly 20:5*n*-3 and 22:6*n*-3, which constitute usually almost half of the total fatty acids [17,28]. In contrast, the nudibranchs did not show this property; these two marine PUFA were minor components and constituted in sum about 2% of the total fatty acids (range 1.4%–7.3%) (Figure 4). Nevertheless, sea slugs exhibited some unique features in their fatty acid composition. Their fatty acid profiles were distinguished drastically from those of other mollusks. The differences seem to be more obvious compared with fatty acids of a common marine snail, *Nucella heyseana*, and limpet, *Acmea pallida* [28] (Figure 4).

**Table 3:** Identification of the 4,4-dimethyloxazoline derivatives and fatty acid composition (wt%) of *Chromodoris michaeli*. Results are expressed as the mean ± SD of four replicates (*n* = 4).

FA	Molecular Ion (*m*/*z*)	% of Total FA	FA	Molecular Ion (*m*/*z*)	% of Total FA
12:0	253	0.4 ± 0.1	20:5*n*-3	355	0.2 ± 0.1
14:0	281	0.9 ± 0.3	20:2Δ5,11	355	1.8 ± 0.5
*iso*-15:0	295	1.9 ± 0.5	20:2Δ5,13	355	1.3 ± 0.4
*anteiso*-15:0	295	0.3 ± 0.1	20:3*n*-6	359	0.7 ± 0.3
15:0	295	1.1 ± 0.2	20:1*n*-11	363	5.6 ± 0.7
*iso*-16:0	309	0.5 ± 0.1	20:1*n*-9	363	0.2 ±0.1
*anteiso*-16:0	309	0.7 ± 0.1	20:1*n*-7	363	2.5 ± 0.6
16:1*n*-7	307	1.7 ± 0.6	21:2Δ7,13	375	0.1 ± 0.0
16:0	309	5.9 ± 0.8	*iso*-21:1	377	0.4 ± 0.1
*iso*-17:0	323	1.4 ± 0.3	21:1*n*-7	377	2.0 ± 0.1
*anteiso*-17:0	323	0.9 ± 0.1	21:1*n*-5	377	1.3 ± 0.5
17:1*n*-8	321	1.4 ± 0.4	22:5*n*-6	383	0.4 ± 0.1
17:1*n*-6	321	0.4 ± 0.1	22:6*n*-3	381	0.7 ± 0.2
17:0	323	1.4 ±0.1	22:4*n*-6	385	10.2 ± 1.3
18:3*n*-6	331	0.2 ± 0.1	22:5*n*-3	383	0.2 ± 0.1
iso-18:0	337	0.4 ± 0.2	22:3*n*-6	387	0.2 ± 0.1
*anteiso*-18:0	337	0.4 ± 0.2	22:2Δ7,13	389	3.6 ± 0.7
18:2*n*-6	333	7.0 ± 0.9	22:2Δ7,15	289	1.2 ± 0.3
18:1*n*-9	335	5.4 ± 0.4	22:1*n*-9	391	0.4 ± 0.2
18:1*n*-7	335	3.4 ± 1.0	22:1*n*-7	391	0.1 ± 0.1
18:0	337	7.4 ± 1.3	*iso*-24:2Δ5,9	417	0.2 ± 0.1
*iso*-19:1	349	0.1 ± 0.0	24:2Δ5,9	417	3.1 ± 0.5
*anteiso*-19:1	349	0.2 ± 0.1	*iso*-25:2Δ5,9	431	4.0 ± 1.0
*iso*-19:0	351	0.2 ± 0.1	*anteiso*-25:2Δ5,9	431	0.6 ± 0.2
*anteiso*-19:0	351	0.2 ± 0.1	25:2Δ5,9	431	0.1 ± 0.1
19:1*n*-8	349	0.1 ± 0.0	*iso*-26:2Δ5,9	445	0.8 ± 0.3
19:1*n*-12	349	2.6 ± 0.8	*anteiso*-26:2Δ5,9	445	0.1 ± 0.0
19:0	351	0.3 ± 0.1	26:2Δ5,9	445	0.8 ± 0.3
20:4*n*-6	357	10.5 ± 1.2	*anteiso*-27:2Δ5,9	459	0.2 ± 0.1

In the nudibranchs, a significant amount of VLCFA specific to sponges, so-called demospongic acids, was found. These nudibranchs are carnivorous and specialized feeders on sponges. Utilization of this food probably is responsible for the high level of the demospongic acids in these mollusks. It is suspected that the majority of sea slugs feed on certain sponge species, which are known to be distinguished in their fatty acid composition [29]. Indeed, a series of VLCFA with double bonds at Δ5,9 positions in the chain was identified in the tropical nudibranchs (Table 4). Concentrations of these components differed among the species. Among VLCFA of *Platydoris* sp., only hexacosadienoic acid 26:2∆5,9 was identified, whereas in *Chromodoris michaeli*, tetracosadienoic 24:2∆5,9 and branched *iso*-25:2∆5,9 were dominant, with some minor VLCFA. Moreover, branched hexacosatrienoic acids, *iso*-26:3∆5,9,19 and *anteiso*-26:3∆5,9,19, were found only in *Glossodoris cincta*, and *iso*-27:2∆5,9 was identified only in *C. michaeli*. The specific distribution of the VLCFA suggests that these nudibranchs may feed on different sponge species.

**Table 4:** The distribution of very long-chain fatty acids of nudibrans according to the degree of unsaturation and chain length (% of total fatty acids). Results are expressed as the mean ± SD of four replicates (*n* = 4).

VLCFA	*Chromodoris* sp.	*C. geometrica*	*C. tinctoria*	*C. michaeli*	*Glossodoris atromarginata*	*G. cincta*	*Risbecia tryoni*	*Platydoris* sp.
*i*-24:2∆5,9	-	-	-	0.2	0.2	-	-	-
24:2∆5,9	1.0	4.7	0.4	3.1	1.1	1.2	1.4	-
24:1	-	0.3	0.1	-	-	-	-	-
*i*-25:2∆5,9	-	-	-	4.0	-	-	-	-
25:2∆5,9	4.0	1.6	0.7	0.2	2.3	2.6	0.5	-
25:3∆5,9	0.5	0.1	-	-	-	1.3	-	-
26:0	-	0.1	-	-	-	0.3	-	-
*i*-26:2∆5,9	0.8	0.1	0.8	0.8	-	0.7		-
26:2∆5,9	6.0	3.0	13.6	0.8	6.3	8.4	5.8	10.5
*i*-26:3∆5,9,19	-	0.2	-	-	1.2	2.3	-	-
*ai*-26:3∆5,9,19	-	-	-		-	1.1	-	-
*ai*-27:2∆5,9	-	-	-	0.2	-	-	-	-

Thus, this study demonstrated that sponges are not the only source of these Δ5,9 dienoic acids, since they were found also in other marine organisms, such as nudibranch mollusks. Various biological activities have been reported to date for the most encountered VLCFA double bonds at Δ5,9 positions in the chain. The use of an antiplasmodial bioassay revealed that fatty acids with 23 to 26 carbon atoms and double bonds in the position Δ5,9 displayed considerable antiprotozoal activity [30]. Thus, these demospongic fatty acids may be the source of very potent antimalarial drugs. These fatty acids are also potent inhibitors of the enzyme, topoisomerase I; this property could lead to the development of effective anti-cancer drugs. The 14-methyl-5,9-pentadecadienoic acid from phospholipids of the gorgonian *Eunicea succinea* was active against Gram-positive bacteria, such as *Staphylococcus aureus* and *Streptococcus faecalis* [31]. The natural compound, 30:3Δ5,9,23, was isolated from the sponge, *Chondrilla nucula*, and was found to be an elastase inhibitor, which is known to be a potential therapeutic agent in various diseases, such as pulmonary emphysema, chronic bronchitis and several inflammatory disorders. In addition, the C23–C26Δ5,9 fatty acids had almost no cytotoxicity on mammalian L6 cells. Therefore, the Δ5,9 FA may be of use against the parasites without damage to the host [32]. Mixtures of the branched 22-methyl-5,9-tetracosadienoic and 23-methyl-5,9-tetracosadienoic acids showed cytotoxic activity against mouse Ehrlich carcinoma cells and a hemolytic effect on mouse erythrocytes [33].

As referenced above, marine mollusks are probably a unique source of unusual unsaturated fatty acids, the non-methylene-interrupted dienoic fatty acids (NMID FA), as opposed to the common methylene-interrupted PUFA, in that their double bonds are separated by more than one methylene group. These ubiquitous and, in some species, major components of the mollusk lipids [28] have been, to date, extensively studied [34]. The NMID FAs were found in all studied species (Figure 3 and Figure 4); among them, common 20:2Δ5,11, 20:2Δ5,13, 22:2Δ7,13 and 22:2Δ7,15 and a novel isomer, 21:2Δ7,13, were identified. FA 21:2Δ7,13 has been reported earlier in other nudibranch species, *Phyllidia coelestis* [18], in the edible bivalve, *Megangulus zyonoensis* [35], and the gonads of the limpets, *Cellana grata* and *Collisella dorsuosa* [36]. The largest concentration of NMID FA was detected in *Risbecia tryoni* (21.5%). The mollusks are able to synthesize the C20 and C22 NMID FA by a Δ5 desaturase acting upon the appropriate precursor, such as 18:1*n*-7 and 18:1*n*-9, and further chain elongation. The potential precursors of 21:2Δ7,13 are 17:1*n*-8 and 19:1*n*-8, which are of bacterial origin and abundant in the nudibranchs (Table 3). It has been recently shown that mollusks expressed a Fad-like gene that encodes an enzyme with Δ5-desaturation activity, which participates in the biosynthesis of NMID FA [37]. Although their biological role and function is not fully understood, it has been suggested that NMID FAs play structural and protective roles in cell membranes, since they are esterified phospholipids and occur in amounts that are often in a reverse relation to 20:5*n*-5 and 22:6*n*-3 [18,34]. The unusual double bond positions in NMID FAs are considered to confer to cell membranes a higher resistance to oxidative processes and microbial lipases than the common PUFA [34]. The introduction of a *trans*-5 double bond into the linoleic acid, 18:2*n*-6, molecule provides a fatty acid, columbinic acid, 18:3Δ5*trans*,9*cis*,12*cis*, with fascinating biological properties [38]. In experiments with essential fatty acid-deficient rats, it has been shown that columbinic acid is effective in maintaining the proper epidermal layer and improves the fertility of the rats, while the inhibition of prostaglandin synthesis has a beneficial effect, since inflammation and the thrombotic tendency are reduced.

Another unique feature of the nudibranchs is associated with an aberrant level of the odd-chain and branched fatty acids (OBFA) that are specific for bacteria and usually named “bacterial fatty acids” (Figure 3). They are normally minor metabolites in most animals, but a high abundance of bacterial acids found in the nudibranchs was extraordinary. The sum of OBFA, predominantly 15:0, 17:0, 17:1*n*-8 and *iso*- and *anteiso*-C15, C16, C17, C18 and C19 fatty acids, reached up to 15.8% in the *Chromodoris geometrica* and 17.4% in *Glossodoris atromarginata*, whereas their concentration in *Platydoris* sp. was the lowest (5.0%). A high level of bacterial fatty acids in the nudibranchs may serve as an indicator that the symbiotic bacteria provide the host with nutrients. Earlier, an abundance of OBFA discovered in *Dendrodoris nigra* allowed us to suggest that symbiotic bacteria may be their source in this nudibranch; transmission electron microscopy (TEM) confirmed the presence of symbiotic bacteria in the cytoplasm of the epithelial cells and the glycocalyx layer covering the epithelium of the notum and the mantle of *D. nigra* [39]. It was found that the bacteria in the glycocalyx sometimes undergo destructive lysis, with their components being utilized by the epithelial cells. The high concentration of typical bacterial fatty acids in the lipids of the nudibranch *D. nigra* agrees well with the results of TEM and confirms that the lysed bacterial cells are utilized by the mollusk tissues [39]. Moreover, some bacterial OBFA, such as 17:1*n*-8 and 19:1*n*-8, evidently serve as potential precursors for the biosynthesis of odd-chain PUFA identified in the nudibranchs, such as 21:2Δ7,13, as well as 21:4*n*-7 isolated from other marine opisthobranch mollusk, *Scaphander lignarius*, and possessed activity against a range of human cancer cell lines (melanoma, colon carcinoma and breast carcinoma) [40].

There is increasing evidence that microbial symbionts are the true source of biologically-active compounds isolated from some species of chemically-rich invertebrates, mainly sponges, bryozoans, isopods and tunicates [41,42]. The symbionts are reported to be producers of the host’s secondary metabolites that have defensive and protective functions for their hosts [43,44]. Many biologically active compounds, including toxic and deterrent secretions, have been isolated from nudibranchs [45]. Interestingly, endobacterial morphotypes have been recently described for twelve of thirteen species of nudibranchs tested [46]. Moreover, the epithelium of the temperate nudibranch, *Rostanga alisae*, rich in OBFA, demonstrated high numbers of symbiont-containing cells (*i.e.*, bacteriocytes) [47]. Taken all together, these results suggest that symbiotic bacteria might be involved in the defense against predators and, so, in production of the bioactive compounds.

## 3. Experimental Section

### 3.1. Site and Samples

Eight species of nudibranchs, *Chromodoris* sp., *C. geometrica*, *C. tinctoria*, *C. michaeli*, *Glossodoris atromarginata*, *G. cincta*, *Risbecia tryoni* and *Platydoris* sp. (phyla: Mollusca, Class: Opisthobranchia; orders: Nudibranchia, Suborder: Doridina) were collected from the Research Vessel Akademik Oparin by SCUBA divers in Nha Trang Bay of the South China Sea, Vietnam, in January 2005, October 2006, June 2007, and April–May 2013. The nudibranchs collected were placed immediately in tanks under water at the site of collection and transported to the laboratory. Three to five specimens of each species were used for lipid analysis.

### 3.2. Lipid Analysis

Tissues of mollusks were crushed, and total lipids were extracted by homogenization in a chloroform/methanol mixture (1:2, v/v) [48]. Lipid classes were separated by one-dimensional silica gel thin-layer chromatography (TLC). The Merk Kieselgel 60 G plates (6 cm × 6 cm) were first developed in hexane/diethyl ether/acetic acid (80:20:1, v/v) to resolve nonpolar compounds. After development, the TLC plates were dried under air flow and developed to 20% length in a polar solvent system of chloroform/acetone/methanol/acetic acid/water (50:20:10:10:1, v/v). Lipids were detected on the TLC plates using 10% H_2_SO_4_/methanol with subsequent heating to 180 °C. The TLC plates were scanned using an image scanner (Epson Perfection 2400 Photo) in grayscale mode. Lipid class concentrations were based on band intensity using an image analysis program (Sorbifil TLC Videodensitometer). Units were calibrated using standards for each lipid class.

Polar lipids were separated by two-dimensional silica gel TLC in the solvent systems: chloroform/methanol/28% NH_4_OH, 65:25:4, v/v, for the first direction; chloroform/acetone/methanol/acetic acid/water, 50:20:10:10:1, v/v, for the second one. Lipids were detected on TLC pales using 10% H_2_SO_4_/methanol with heating to 180 °C and by specific reagents for phospholipids [49], amino-containing lipids (0.5% ninhydrin in waterlogged butanol) and choline lipids (Dragendorff’s reagent). Phospholipids were quantified with the molybdenum reagent [49].

### 3.3. Fatty Acid Analysis

Fatty acid methyl esters (FAME) were prepared by a sequential treatment of the total lipids with 1% sodium methylate/methanol and 5% HCl/methanol in a screw-capped vial [50] and purified by preparative silica gel TLC using benzene as a solvent. 4,4-Dimethloxazoline (DMOX) derivatives were prepared from FAME [51]. The GC analysis of FAME was carried out on a Shimadzu GC-2010 chromatograph (Kyoto, Japan) with a flame ionization detector on a SUPELCOWAX (Supelco, Bellefonte, PA, USA) capillary column (30 m × 0.25-mm internal diameter, 0.25-μm film thickness) at 210 °C. Helium was used as a carrier gas at a linear velocity of 30 cm s^−1^ (the split ratio was 1:30). Injector and detector temperatures were 250 °C. Fatty acids were identified by a comparison with authentic standards and equivalent chain length values (ECL) [52]. Identification of fatty acids was confirmed by gas chromatography-mass spectrometry (GC-MS) of their methyl esters and DMOX derivatives. The GC-MS analysis of FAME was performed on a model Shimadzu GCMS-QP5050A (Kyoto, Japan) fitted with a Supelco MDN-5S capillary column (30 m × 0.25 mm i.d. Supelco, Bellefonte, PA, USA). Ionization of the samples was performed by an electron impact at 70 eV. The column temperature was programmed from 170 °C, held for 1 min, followed by an increase to 240 °C at a rate of 2 °C min^−1^ and then held for 20 min. The temperature of the injector and detector was 250 °C. GC-MS of DMOX derivatives was performed using the same instrument at a column temperature of 210 °C with a 3 °C min^−1^ increase to 270 °C, which was held for 40 min. The injector and detector temperatures were 300 °C. Spectra were compared with the NIST library and fatty acid mass spectra archive [53].

### 3.4. Statistical Analysis

Difference in the mean of lipid concentrations was examined with a one-way ANOVA. In all cases, statistical significance was indicated by *p* < 0.05. All data were expressed as mean ± SD.

## 4. Conclusions

Mollusks, as well as the invertebrates in general, constitute a source of lipid bioactive compounds offering a variety of activities. This study has demonstrated for the first time that nudibranchs exhibit a wide diversity of lipids that differed greatly from that of other marine gastropods. Lipids of nudibranchs were composed mainly of phospholipids rich in plasmalogen PE and plasmalogen PS. The nudibranchs exhibited some unique features in their fatty acid composition. They displayed large amounts of VLCFA, various NMID FAs and a high abundance of OBFA. Many of these fatty acids originate in nudibranchs from unusual biosynthetic pathways, specific dietary sources and symbiotic partnerships with bacteria. The results of this study and of previous research suggest that symbiotic bacteria may play an important role in producing bioactive chemicals or their precursors within the host. The current study has shown that these mollusks may be an important resource of a wide range of bioactive compounds.

## Abbreviations

CAEP: ceramide-aminoethylphosphonate
DPG: diphosphatidylglycerol
FA: fatty acid
FAME: fatty acid methyl ester
FFA: free fatty acids
GC-MS: gas chromatography-mass spectrometry
MADAG: monoalkyldiacylglycerol
MUFA: monounsaturated fatty acid
NMID FA: non-methylene-interrupted dienoic fatty acids
OBFA: odd-chain and branched fatty acids
PC: phosphatidylcholine
PE: phosphatidylethanolamine
PI: phosphatidylinositol
PL: phospholipids
PS: phosphatidylserine
PUFA: polyunsaturated fatty acid
SFA: saturated fatty acid
ST: sterols
TLC: thin-layer chromatography
VLCFA: very long chain fatty acid
